# Simultaneous detection of nucleotide excision repair events and apoptosis-induced DNA fragmentation in genotoxin-treated cells

**DOI:** 10.1038/s41598-018-20527-6

**Published:** 2018-02-02

**Authors:** Soyun Baek, Sueji Han, Dukjin Kang, Michael G. Kemp, Jun-Hyuk Choi

**Affiliations:** 1 Center for Bioanalysis, Korea Research Institute of Standards and Science, Daejeon, Republic of Korea; 2 Department of Bio-Analytical Science, University of Science & Technology, Daejeon, Republic of Korea; 3 Department of Pharmacology and Toxicology, Wright State University Boonshoft School of Medicine, Dayton, Ohio USA

## Abstract

Novel *in vivo* excision assays for monitoring the excised oligonucleotide products of nucleotide excision repair in UV-irradiated cells have provided unprecedented views of the kinetics and genomic distribution of repair events. However, an unresolved issue is the fate of the excised oligonucleotide products of repair and their mechanism of degradation. Based on our observation that decreases in excised oligonucleotide abundance coincide with the induction of apoptotic signaling in UV-irradiated cells, we considered the possibility that caspase-mediated apoptotic signaling contributes to excised oligonucleotide degradation or to a general inhibition of the excision repair system. However, genetic and pharmacological approaches to inhibit apoptotic signaling demonstrated that caspase-mediated apoptotic signaling does not affect excision repair or excised oligonucleotide stability. Nonetheless, our assay for detecting soluble DNAs produced by repair also revealed the production of larger DNAs following DNA damage induction that was dependent on caspase activation. We therefore further exploited the versatility of this assay by showing that soluble DNAs produced by both nucleotide excision repair and apoptotic signaling can be monitored simultaneously with a diverse set of DNA damaging agents. Thus, our *in vivo* excision repair assay provides a sensitive measure of both repair kinetics and apoptotic signaling in genotoxin-treated cells.

## Introduction

The human nucleotide excision repair system removes a wide variety of lesions from DNA through a dual incision mechanism, in which the damaged nucleotides are essentially cut out of the DNA in the form of a small DNA oligonucleotide approximately 30 nt in length^1,2^. Though this phenomenon was initially demonstrated *in vitro* using defined DNA damage-containing templates and cell-free extracts^3,4^ or purified repair proteins^5,6^, nearly identical observations have subsequently been made using cultured cells exposed to UV or other related environmental carcinogens and anti-cancer compounds. The detection of these repair events *in vivo* was made possible by the fact that these excised oligonucleotides are readily solubilized during cell lysis, and, once purified, can be labeled, separated by electrophoresis, and detected via radioactivity or chemiluminescence^7–10^. Moreover, the fact that these excised DNAs are released from DNA in association with specific repair proteins^7,11^ has allowed for studies of post-excision repair events^12,13^ and for the mapping of DNA repair events throughout the genome^2,14–18^.

These assays for monitoring excision repair capture only a transient repair intermediate in nucleotide excision repair. Ultimately, the 30-nt-long, damage-containing excised oligonucleotides are degraded to smaller species that must be detected via other methods^19^. How these repair products are degraded is completely unknown^13,20^, but their efficient degradation and intracellular processing are likely important to general cell biology and to human health. Indeed, the aberrant localization and processing of DNA in the cytosol leads to the activation of innate immune pathways implicated in autoimmunity^21–24^, and mutations in DNases responsible for degrading different DNA species cause autoimmune disorders such as lupus erythematosus in both experimental animal models and human patients^25–30^. Defective processing of the excised oligonucleotide products of repair or of other DNA species may therefore contribute to human disease, and thus their mechanism of degradation needs to be characterized and understood.

Based on our observation that the induction of apoptotic signaling was correlated with a decrease in excised oligonucleotide abundance in cultured cells following UV exposure, we tested the hypothesis that nucleotide excision repair and/or excised oligonucleotide stability were directly regulated by caspase-mediated apoptotic signaling. Though our analyses showed no strong link between caspase signaling and repair inhibition, our assays for monitoring excision repair unexpectedly showed that caspase-dependent degradation of genomic DNA can be readily and simultaneously observed along with bona fide excision repair events. Thus, this discovery demonstrates the versatility of our assay for monitoring soluble DNAs that are generated in genotoxin-treated cells and should therefore facilitate future studies of DNA damage responses in human cells exposed to a wide variety of DNA damaging agents.

## Materials and Methods

### Cell culture and genotoxin exposure

HeLa cells were obtained from the Korean Cell Line Bank of Seoul National University (Seoul, Korea). Cells were cultured in Dulbecco’s modified Eagle’s medium (Gibco) supplemented with 10% fetal bovine serum at 37 °C in a 5% CO_2_ humidified incubator. For UV exposure, cells were washed with phosphate-buffered saline (PBS, Gibco) and then exposed to the indicated doses of UV using a germicidal lamp that emits primarily 254 nm light. The intensity of radiation was determined by using a portable digital radiometer (UVX Digital radiometer UVP). Benzo[a]pyrene-7,8-dihydrodiol-9,10-epoxide (BPDE) was obtained from the NCI Chemical Carcinogen Reference Standard Repository (Midwest Research Institute, MO). Cisplatin and camptothecin were purchased from Sigma. For treatment of these DNA-damaging agents, the indicated doses of each chemical compound were directly added to the culture medium at the indicated concentrations.

### Caspase inhibition

Caspase inhibition was achieved by using Z-VAD-FMK (Promega) in the culture medium at 20 μM unless otherwise indicated. For siRNA-mediated knockdown of Caspase 3, siGENOME human Casp3 (836) siRNA SMARTpool (Dharmacon) was transfected into cells using DharmaFECT 1 transfection reagent (Dharmacon) according to the manufacturer’s instructions. As a negative control for siRNA experiments, siGENOME Non-Targeting siRNA pool #1 (Dharmacon) was used.

### Immunoblotting

Proteins were analyzed on NuPAGE 4–12% Bis-Tris Protein Gels (Invitrogen) and subjected to immunoblotting analysis. Antibodies used for immunoblotting included anti-cleaved PARP (#9546), anti-cleaved Caspase-3 (#9661), anti-GAPDH (#2118), anti-XPC (sc74410), anti-XPA (sc-853) from Santa Cruz Biotechnology, and anti-RPA32 (NA18, Calbiochem), anti-p62 (sc-292), anti-p89 (sc-293), anti-Caspase-3 (#9665) from Cell Signaling Technology. Secondary antibodies included horseradish peroxidase-linked anti-mouse (sc-2005) and anti-rabbit IgG (sc-2004) from Santa Cruz Biotechnology. Chemiluminescence was visualized with Amersham ECL Western or Prime Western Blotting Detection Reagent (GE Healthcare) on an ImageQuant LAS 4000 Mini apparatus (GE Healthcare) and quantified using ImageQuant TL Software (GE Healthcare). The specificity of all commercially available antibodies used for immunoblotting was verified with appropriate size markers, and the graphed data validates the reproducibility our findings in at least two independent experiments.

### Immunoslot blot assay

Genomic DNA was isolated from insoluble pellets obtained during the preparation of cell lysates and subjected to immunoslot blot analysis as described previously^9,31^.

### Cell lysis and ***in vivo*** excision assay

Genotoxin-treated cells were washed with ice-cold PBS and harvested with a cell scraper at the indicated time points. After centrifugation (1,000 × g), the cell pellets were resuspended in a Triton X-100 lysis buffer (20 mM Tris-HCl, pH 7.5, 150 mM NaCl, 1 mM EDTA, 1 mM EGTA, and 1% Triton X-100) and incubated for 15 min at 4 °C on a rotating mixer. Soluble cell lysates were prepared by centrifugation (20,000 × g) for 1 h at 4 °C and transferred to new tubes. Small portions of the lysates were saved for immunoblot analysis. The cell lysates were incubated with RNase A (10 μg/ml) and RNase T1 (20 units/ml) for 20 min at 37 °C, and then treated with 0.15 mg/ml of proteinase K for 30 min at 55 °C. After phenol-chloroform extraction and ethanol precipitation, the extracted DNA samples were resuspended in TE buffer, and then subjected to labeling with terminal deoxynucleotidyl transferase (TdT) and the nucleotide analog biotin-11-dUTP as described previously^8,9^. Briefly, the resuspended DNA was 3′-end labeled with 2 μM of biotin-11-dUTP (Biotium, Inc.) and 0.2 units/μl of TdT (New England Biolabs) in 1X TdT reaction buffer supplemented with 0.25 mM CoCl_2_ for 30 min at 37 °C. The labeling reactions were stopped by addition of 10 mM EDTA. About 100 ng of 10 bp DNA ladder (Invitrogen) was labeled in an identical reaction for use as a DNA marker during gel electrophoresis. The reactions were then treated with RNase A (10 μg/ml) and RNase T1 (20 units/ml) for 20 min at 37 °C and incubated with 0.4% SDS and 40 μg/ml of proteinase K for 30 min at 55 °C. After phenol-chloroform extraction and ethanol precipitation, the labeled DNA samples were separated on 10–12% TBE-urea gels, transferred onto a modified nylon membrane (Zeta-Probe membrane by Bio-Rad or Biodyne B membrane by Pierce), and then fixed by UV crosslinking. The membrane was incubated with 1X PBS containing 2% SDS for 30 min and incubated with streptavidin-HRP conjugates (Thermo Scientific) in 1× PBS containing 2% SDS for 30 min. The membrane was then washed three times for 10 min with 1X PBS containing 0.5% SDS and incubated with 200 mM Tris-HCl (pH 9.0) containing 10 mM MgCl_2_ for 5 min. Chemiluminescence was visualized with Amersham ECL Western or Prime Western Blotting Detection Reagent (GE Healthcare) on ImageQuant LAS 4000 Mini apparatus (GE Healthcare) and quantified by ImageQuant TL Software (GE Healthcare). Detection of biotin-labeled DNA could also be achieved with the Chemiluminescent Nucleic Acid Detection Module (Thermo Scientific), according to the manufacturer’s instructions. The entire blot was probed with streptavidin-HRP conjugates, and the entire image is displayed in each of the figures along with longer exposures of the region of the gel corresponding to the excised products of repair. To determine the size of DNA fragments, 10 bp DNA ladder (Invitrogen) was used.

### Immunoprecipitation of excised oligomers

Detection of excised oligomers containing specific UV photoproducts was performed as previously described^7–9^.

## Results

### Nucleotide excision repair and apoptotic signaling show distinct kinetics in UV-irradiated cells

**Figure 1:**
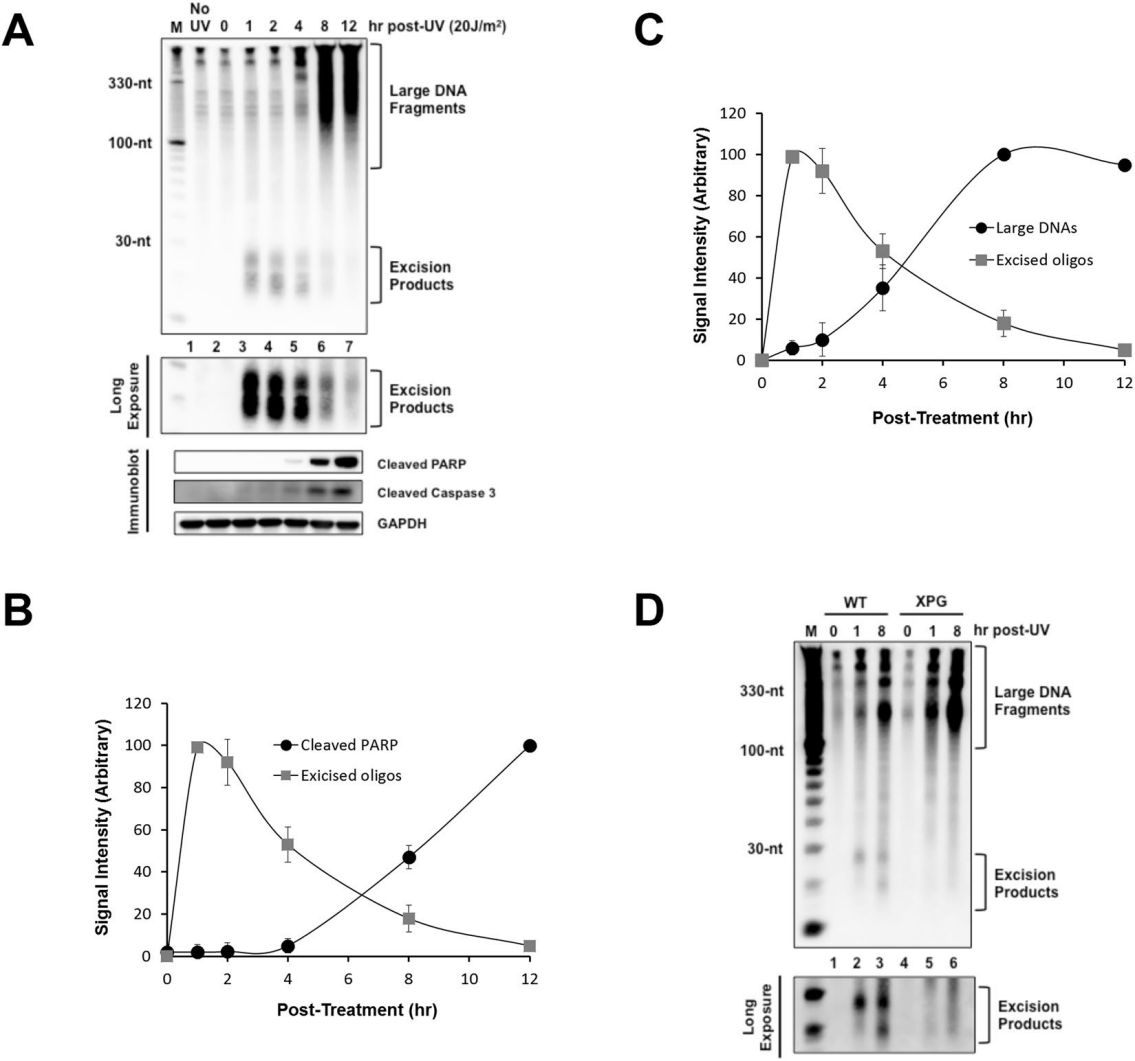
Correlation between apoptotic signaling and the generation of large DNAs with decreased generation of excised oligonucleotide repair products. (**A**) Analysis of large DNA fragments and excised oligomers released from genomic DNA following UV irradiation. HeLa cells were exposed to 20 J/m^2^ of UVC and incubated for the indicated time points. After extraction of low molecular weight of DNAs from the cells, the DNA molecules were labeled with biotin at 3′-end using terminal deoxynucleotidyl transferase (TdT). The labeled DNA molecules were separated by gel electrophoresis, transferred to a nylon membrane and immobilized by UV cross-linking. The membrane was then incubated with HRP-conjugated streptavidin, and the labeled DNA molecules were detected with a chemiluminescence reagent (upper panel). A small portion of the UV-irradiated cells were lysed and analyzed by SDS-PAGE and immunoblotting with the indicated antibodies. (**B**) Quantification of the excised oligonucleotide repair products and cleaved PARP signals as shown in (**A**). The relative signal intensities were determined by setting the highest signal to 100. The results were quantified and are plotted as mean and standard deviations. (**C**) Quantitative analysis of the excised repair products and the large DNA molecules. (**D**) Wild-type (AA8) and XPG mutant (UV135) CHO cell lines were exposed to UV and processed as described above for the detection of soluble DNAs.

The human nucleotide excision repair system responds to UV-induced DNA damage by removing UV photoproducts from the genome in the form of short DNA oligonucleotides approximately 30 nt in length both *in vitro*^3–5^ and *in vivo*^7,8,14^. These primary repair products, as well as secondary, partially degraded products approximately 18- and 24-nt in length, are released from duplex DNA bound to specific DNA repair proteins^11,32^ and can be readily extracted from UV-irradiated cells and separated from bulk genomic DNA by using a mild lysis buffer and high-speed centrifugation^8,9^. The small, soluble repair products are then purified, labeled on the 3′ end with a biotinylated nucleotide, and electrophoresed on a sequencing gel. Following electrophoretic transfer to a nylon membrane, the DNAs can be visualized with streptavidin-bound HRP and chemiluminescent detection^10^. An example of this repair assay in HeLa cells exposed to 20 J/m^2^ of UVC and then harvested at various time points following irradiation is shown in Fig. 1A (top panel).

As previously reported, the peak generation of these excised oligonucleotide repair products occurs 1 h following UV exposure^8,9^. At later time points, the steady state level of the excised oligonucleotides decreases, such that by 8–12 h post-UV, the excised oligonucleotide products of repair are barely visible. The low level of repair products at these time points is not due to the completion of UV photoproduct repair, however, because immunoslot blot analysis of the genomic DNA from these cells revealed that more than 60% of UV-induced CPDs remain in genomic DNA as unrepaired potential substrates for the nucleotide excision repair machinery 8 to 12 h after UV exposure. Whether the low level of repair products that are present at these time points is due to a lower rate of damage excision or to an increased rate of excised oligonucleotide degradation is not known.

Nonetheless, the large fluence of UV used in this experiment (20 J/m^2^) is known to saturate nucleotide excision repair capacity^8^ and to significantly compromise cell viability. Because caspase-mediated apoptotic signaling is a major mechanism by which UV induces cell death^33^, we therefore also examined the induction of apoptotic signaling in the same cell lysates that were used to monitor excision repair. As shown in Fig. 1A (bottom), cleavage of two well-known apoptotic markers (PARP and caspase 3) was observed 8–12 h post-UV, which corresponds to the same time points at which the excised oligonucleotides become barely visible. Quantitation of excision repair and PARP cleavage from several independent experiments revealed that the increase in apoptotic signaling was correlated with a decrease in excised oligonucleotide abundance (Fig. 1B).

Interestingly, examination of soluble DNAs from the UV-irradiated cells also revealed the emergence of a larger species of DNAs that began at the origin of the gel and smeared down to a size of approximately 70 nt in length (Fig. 1A, top). The presence of these DNAs in the cell lysates coincided in time with the activation of apoptotic signaling and with the decrease in excised oligonucleotides (Fig. 1B,C). Importantly, these large DNAs were also observed in UV-irradiated cells that are deficient in nucleotide excision repair (Fig. 1D), which demonstrates that the nucleotide excision repair system is not responsible for generating these large, soluble DNA fragments.

**Figure 2:**
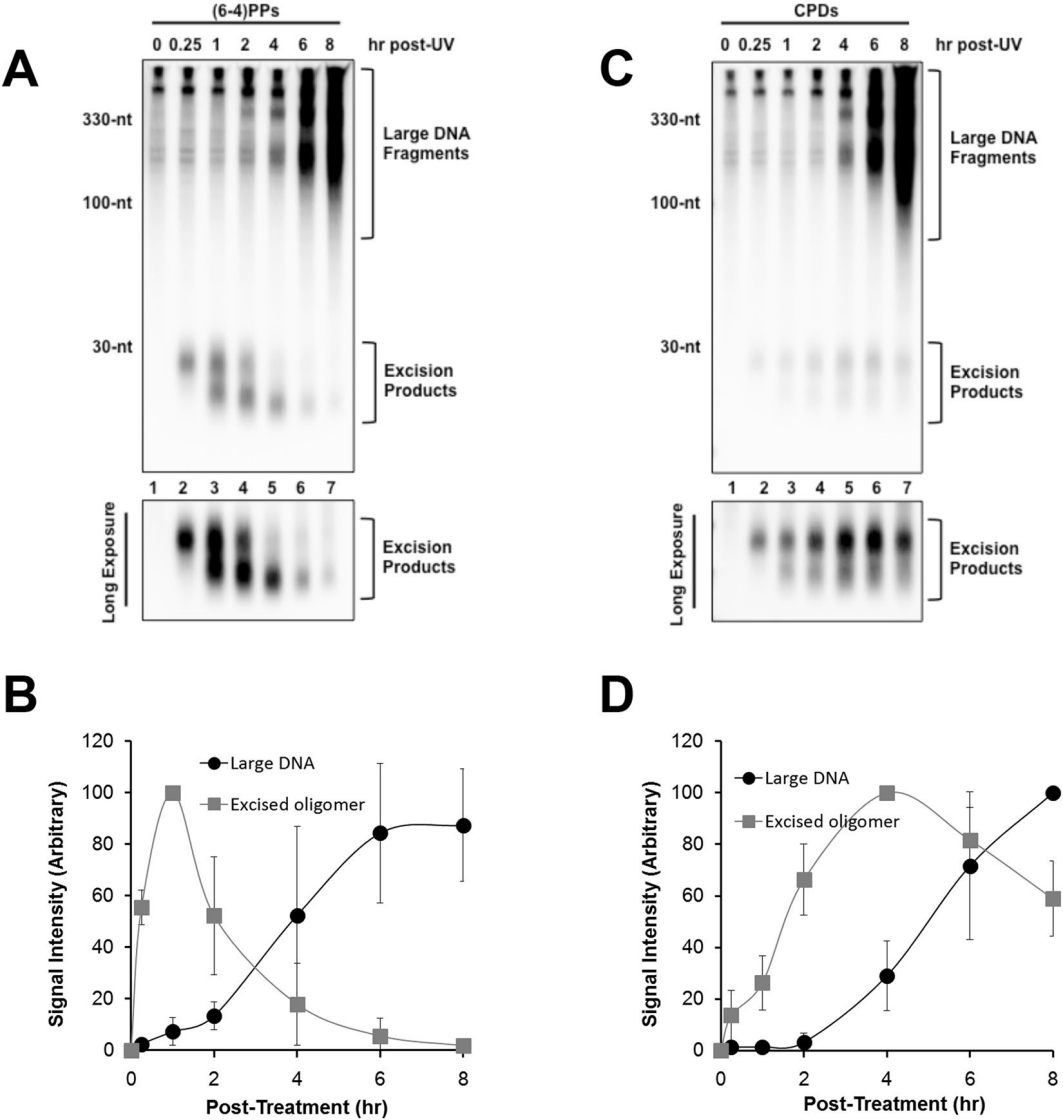
Emergence of large DNA fragments containing (6-4)PPs and CPDs at later time points with decreased generation of excision repair products. UV-irradiated HeLa cells were harvested at the indicated time points and subjected to extraction of soluble DNA fragments released from genomic DNA. The extracted DNA molecules were then immunoprecipitated with anti-(6-4)PPs (**A**) or anti-CPDs antibodies (**C**) and analyzed as described in Fig. 1(A). The relative intensities of the excised oligonucleotides and the large DNA molecules containing (6-4)PPs and CPDs were quantified and shown in (**B**) and (**D**), respectively.

The analysis of excised oligonucleotides shown in Fig. 1A represents the totality of diverse excision products that are generated by the nucleotide excision repair system following UV exposure. To determine whether the kinetics of repair of specific UV photoproducts exhibit a similar inverse correlation with apoptotic signaling, we used anti-(6-4)PP and anti-CPD antibodies to immunoprecipitate soluble DNAs containing the corresponding specific DNA lesions. As shown in Fig. 2A, (6-4)PP-containing repair products are generated within minutes following UV exposure, peak 1 h after UV irradiation, and then begin to rapidly decrease in abundance. However, examination of the top of the gel revealed larger (6-4)PP-containing DNAs that became apparent at late time points after UV exposure. Quantitation of several independent experiments confirmed the opposing kinetics of the excision repair products and large DNAs (Fig. 2B). Though CPDs are recognized less efficiently than (6-4)PPs^34^ and thus are removed from the genome at a slower rate, we noted a similar pattern of large and small DNAs containing CPDs. Thus, large CPD-containing DNAs become more abundant at late time points after UV as the canonical CPD-containing repair products became less abundant (Fig. 2C,D).

Thus, we conclude that the generation and/or stability of the excised oligonucleotide products of nucleotide excision repair show an opposite pattern in comparison with apoptotic signaling and the generation of large, soluble, UV photoproduct-containing DNAs. The activation of apoptosis is therefore correlated with a reduction in the excised oligonucleotides and the emergence of a new, larger soluble DNA species. The fact that these large DNAs contain bona fide UV photoproducts suggests that DNA fragments containing UV photoproducts are released from bulk genomic DNA to a some extent in dying cells.

### Nucleotide excision repair and apoptotic signaling show different kinetics in cells exposed to UV mimetics

The nucleotide excision repair system acts on DNA adducts induced by a wide variety of agents, including bulky aromatic lesions induced by the environmental carcinogen BPDE^35–37^ and intrastrand crosslinks caused by the anti-cancer drug cisplatin^38–41^. Because we previously showed that excised oligonucleotide products of repair could be observed in cells treated with these compounds^9^, we next quantified the level of excised oligonucleotides, cleaved PARP, and large, soluble DNAs as a function of time in cells treated with BPDE and cisplatin. As shown in Supplementary Figure 1, we show that maximal abundance of the excision repair products occurred prior to the generation of cleaved PARP and the large DNAs, which became more abundant as the repair product signal began to decrease. We conclude from these experiments that multiple DNA damaging agents that generate substrates for the nucleotide excision repair system and induce apoptosis also cause the formation of large, soluble DNAs that are generated coincident with apoptotic signaling. Moreover, a decrease in either repair rate or excised oligonucleotide stability of these diverse forms of DNA damage is correlated in time with the activation of caspase-mediated apoptotic signaling.

### Apoptotic signaling does not affect excision repair rate, excised oligonucleotide stability, or excision repair protein levels

**Figure 3:**
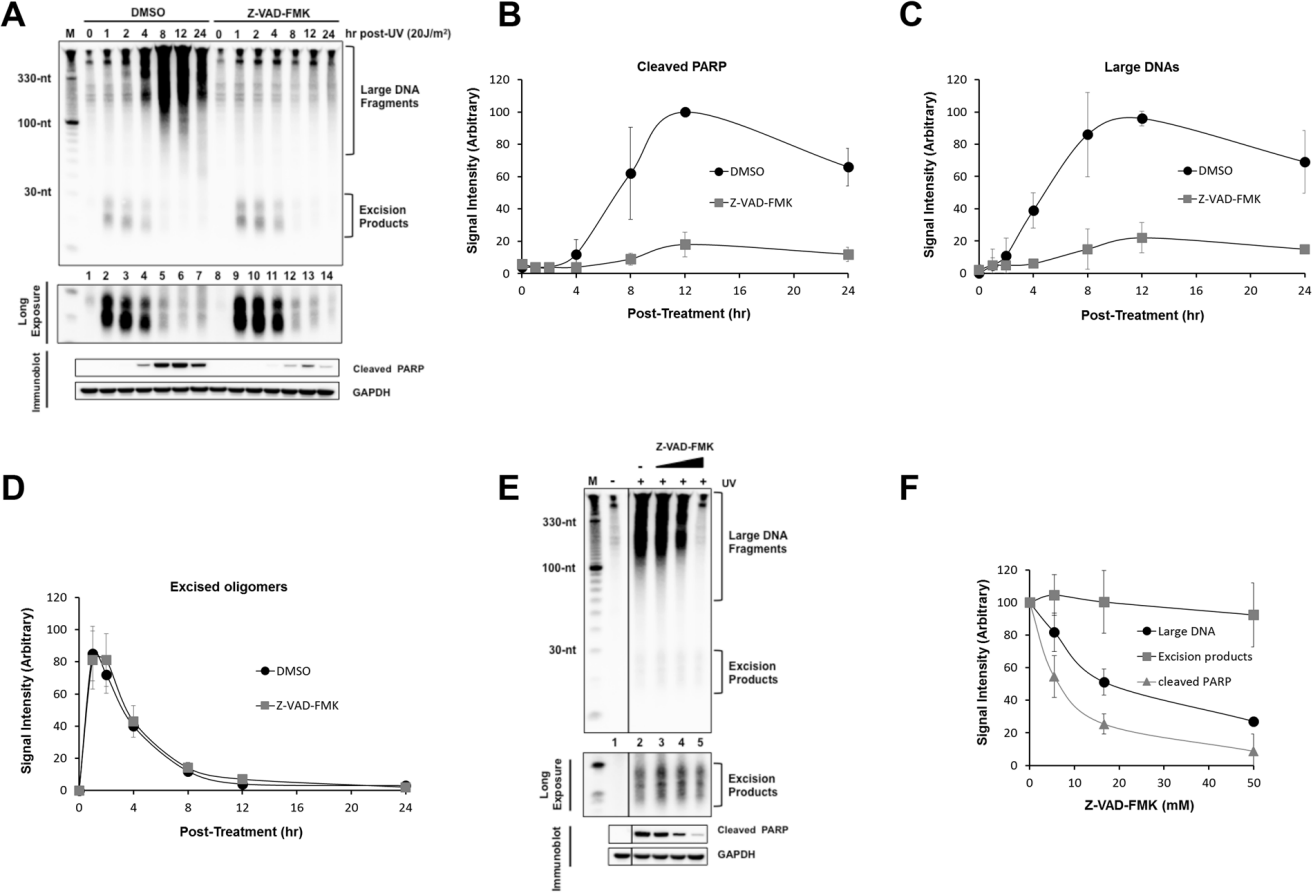
Caspase inhibition prevents PARP cleavage and generation of large DNA fragments but does not affect excision repair activity. (**A**) Effects of caspase inhibition on the generation of large DNA fragments and excised oligomers released from genomic DNA following UV irradiation. HeLa cells were pre-incubated with DMSO or the caspase inhibitor Z-VAD-FMK (20 μM) for 1 h and then exposed to 20 J/m^2^ of UVC. The UV-irradiated cells were further incubated, harvested at the indicated time points, and analyzed for generation of large DNAs and excised oligonucleotides released from genomic DNA. Levels of cleaved PARP (**B**), generation of large DNAs (**C**) and excised oligos (**D**) were quantified and are presented as the mean values ± standard deviation. (**E**) Dose-dependent inhibition of caspase. DMSO or Z-VAD FMK (20 μM)-treated HeLa cells were exposed to 20 J/m^2^ of UVC, harvested 8 h later and analyzed for the generation of large DNAs, excised oligomers. The image was cropped in order to place a non-irradiated negative control adjacent to the UV-treated samples. (**F**) Levels of cleaved PARP, generation of large DNAs and excised oligos in (**E**) were quantified and are presented as the mean values ± standard deviation.

Given that apoptotic signaling is a highly coordinated enzymatic process that maintains cellular energy supplies during cell death^42,43^, the notion that apoptotic signaling could essentially “turn off” nucleotide excision repair to avoid wasting cellular energy would appear to be a reasonable hypothesis. To test this idea, we therefore pre-treated cells with DMSO or the pan-caspase inhibitor Z-VAD-FMK prior to exposure to a high dose of UV. As shown in Fig. 3A and quantified in Fig. 3B, this treatment greatly reduced the amount of cleaved PARP that was present in the cells following the UV exposure. Interestingly, we also noted that the caspase inhibitor significantly abrogated the generation of the large, soluble DNA species that are generated in cells at late time points following UV irradiation (Fig. 3A,C). This result demonstrates that these large DNA species are not a result of simple DNA breaks produced by general DNA metabolism that undergo non-specific nucleolytic processing. Rather, the fact that caspase inhibition blocks their production shows that these DNAs are specifically generated in a caspase-dependent manner.

In contrast, and somewhat unexpectedly, the level of excised oligonucleotides that were present in the UV-irradiated cells was not affected to any significant extent by caspase inhibition (Fig. 3A,D). Moreover, experiments varying the dose of the caspase inhibitor showed that although the use of increasing concentrations of the Z-VAD-FM resulted in a clear reduction in PARP cleavage and the production of large, soluble DNAs, the canonical excision repair products were unaffected (Fig. 3E,F). These results indicate that apoptotic signaling does not have a significant impact on either the generation or stability of the excised oligonucleotide products of nucleotide excision repair.

**Figure 4:**
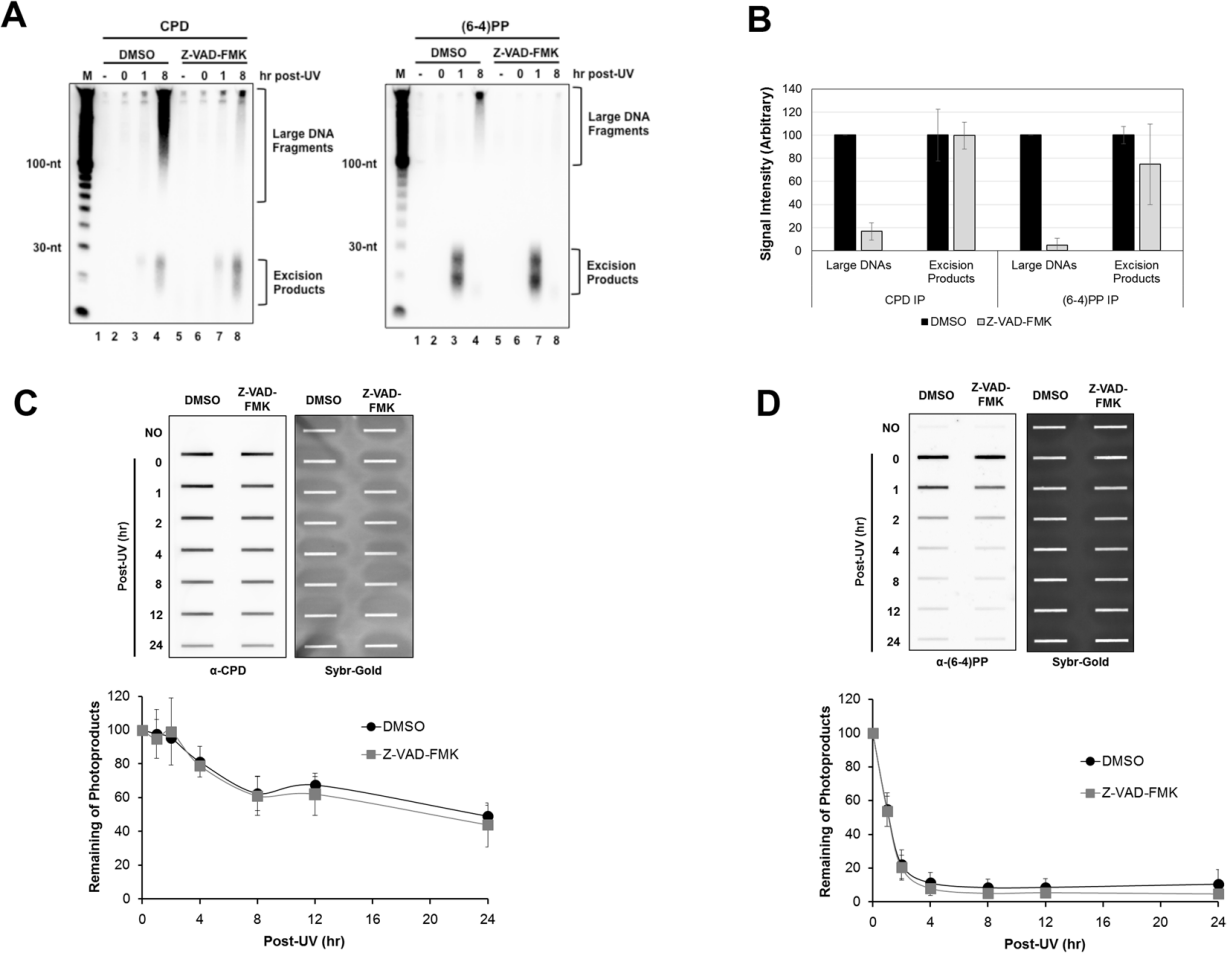
Effects of caspase inhibition on the generation of large DNA fragments containing specific UV photoproducts. (**A**) DMSO or Z-VAD-FMK (20 μM) treated-HeLa cells were exposed to 20 J/m^2^ of UVC, harvested at the indicated time points, and subjected to extraction of soluble DNA fragments. The purified DNAs were immunoprecipitated with anti-CPDs (left) or anti-(6-4)PPs (right) and analyzed as described in Fig. 1(A). (**B**) Quantitative analysis is shown for the levels of large DNAs and excised oligomers. **(C** and **D**) Genomic DNA was isolated from DMSO or Z-VAD-FMK (20 μM) treated-HeLa cells after UV-irradiation and then used in immune-slot blot assays with antibodies against CPDs **(C)** and (6-4)PPs **(D)**. The Sybr-Gold staining shows equal loading of total genomic DNA. Quantitative analyses of repair kinetics for CPDs and (6-4)PPs are shown below the representative images.

As we previously showed that the large DNA species that are present at later time points following UV exposure contain specific UV photoproducts (Fig. 2), we repeated our experiments with UV-irradiated cells treated with the caspase inhibitor but used anti-CPD and anti-(6-4)PP antibodies to isolate specific UV photoproduct-containing DNAs. As shown in Fig. 4A and quantified in Fig. 4B, treatment with the caspase inhibitor largely abrogated the generation of the large damage-containing DNAs but did not significantly impact the generation of the canonical excision repair products.

We next wished to use an independent experimental methodology to confirm our finding that apoptotic signaling does not affect nucleotide excision repair. We therefore used immunoslot blot analysis to monitor the levels of unrepaired UV photoproducts in genomic DNA from UV-irradiated cells that were treated or not with the caspase inhibitor Z-VAD-FMK. As shown in Fig. 4C, approximately 50% of CPDs were removed from genomic DNA by 24 after UV exposure, regardless of the treatment with the caspase inhibitor. Though repaired at a faster rate, (6-4)PP removal was similarly unaffected by caspase inhibition (Fig. 4D). We conclude from these experiments that caspase inhibition does not affect UV photoproduct removal rate or excised oligonucleotide stability.

The caspases that are activated during apoptosis cleave a variety of cellular proteins to abrogate unnecessary biochemical processes and conserve cellular energy for apoptosis^44^. However, our observation that caspase inhibition does not affect nucleotide excision repair rate or excised oligonucleotide stability suggests that nucleotide excision repair proteins may not be targeted by canonical apoptotic caspase signaling. We therefore pre-treated cells with DMSO or the pan-caspase inhibitor Z-VAD-FMK prior to exposure to a high dose of UV and then prepared soluble protein lysates at various time points following irradiation. Though caspase inhibition largely blocked the generation of cleaved PARP, the protein levels of several essential nucleotide excision repair proteins were not significantly affected by either UV or the caspase inhibitor (Supplementary Figure 2). These results therefore further validate our findings that apoptotic signaling does not influence nucleotide excision repair.

### Large, soluble DNAs are a measure of DNA damage-induced apoptotic signaling

**Figure 5:**
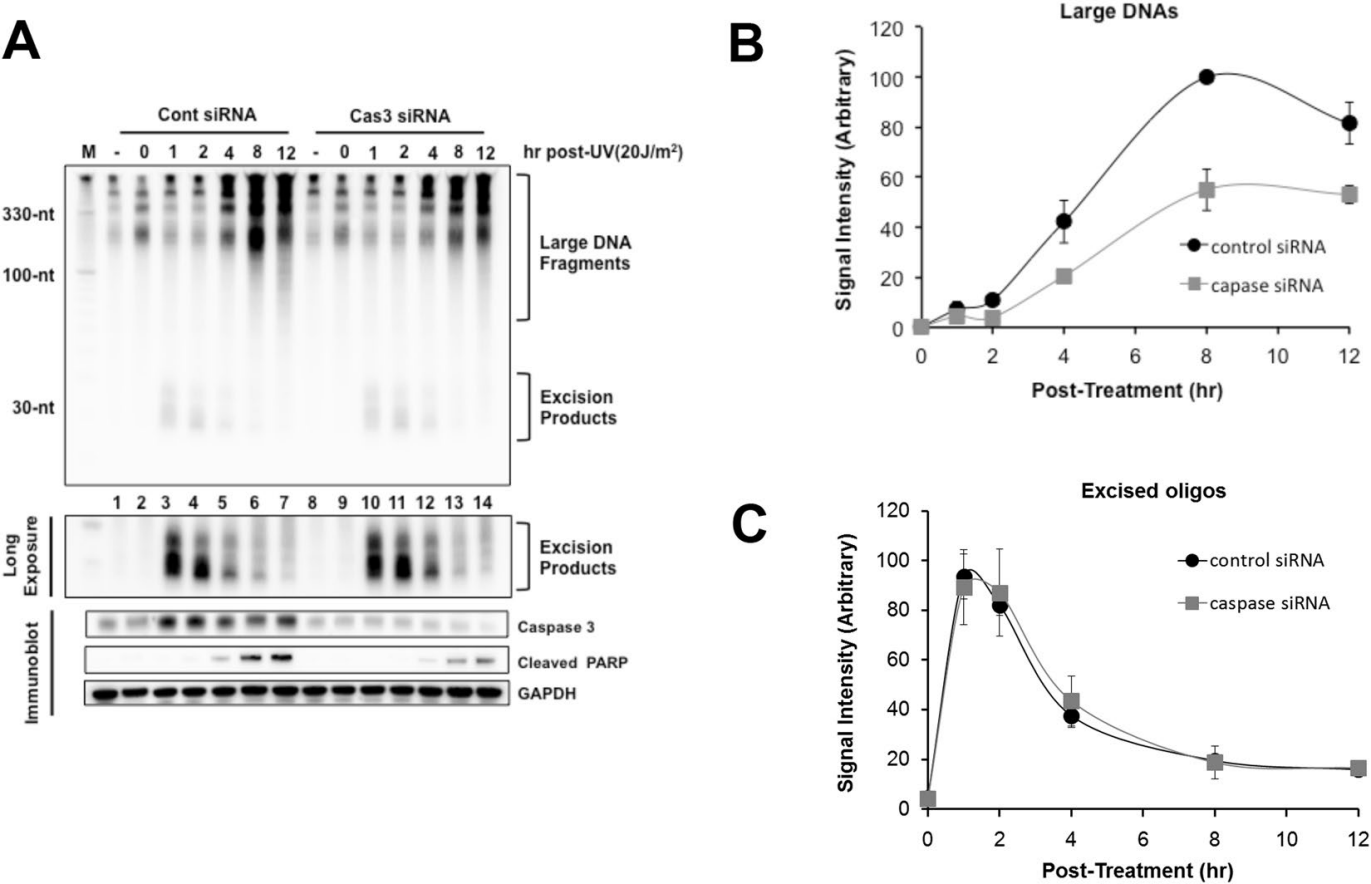
Effects of caspase 3 knockdown on the generation of large DNAs and excised oligomers. **(A)** HeLa cells were transfected with control non-targeting siRNA or caspase 3 siRNA, exposed to 20 J/m^2^ of UVC 48 h later and harvested at the indicated time points. The cells were analyzed for the soluble DNAs (upper panel), and used for immunoblotting with the indicated antibodies (lower panel). Quantitative analysis of the generation of large DNAs **(B)** and excised oligomers **(C)** is also shown.

To confirm our observations that the generation of large, soluble DNAs in UV-irradiated cells could be blocked via the use of a caspase inhibitor, we next used siRNAs to silence the expression of the caspase 3. As shown in Fig. 5A, the siRNAs led to a reduction in UV-induced PARP cleavage. Moreover, the caspase 3 knockdown also partially abrogated the generation of the large, soluble DNA species that appear at late time points following UV exposure (Fig. 5A,B). In contrast, and similar to the results with the pan-caspase inhibitor, the use of the caspase 3 siRNAs did not affect the generation or stability of the small, canonical excised oligonucleotide products of nucleotide excision repair (Fig. 5C). We conclude that although apoptotic signaling does not affect the activity of the nucleotide excision repair system in UV-irradiated cells, it does generate larger soluble DNA species that can readily observed with our method for detecting soluble DNA repair products.

**Figure 6:**
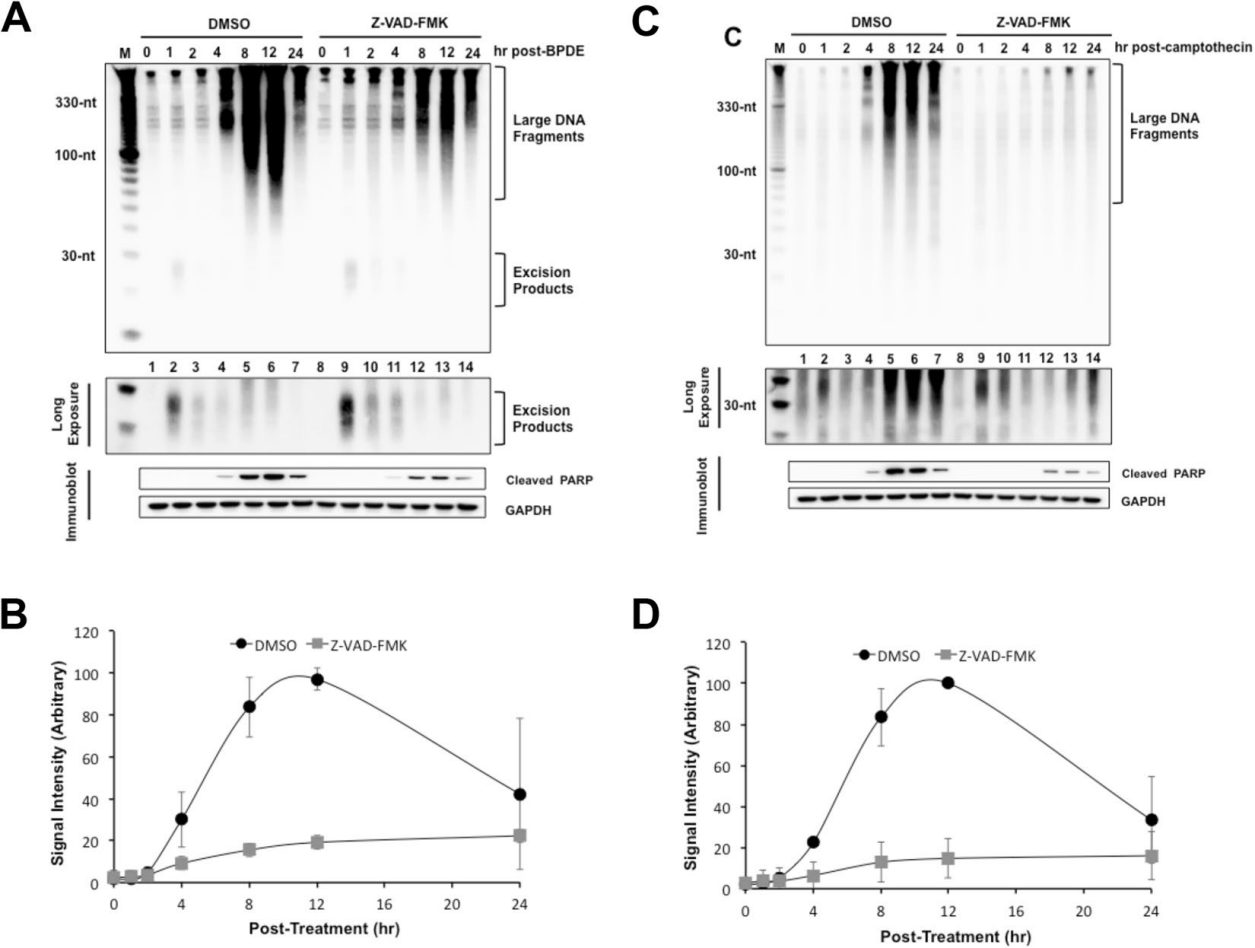
Generation of caspase-dependent large DNA fragments in response to other DNA damaging agents. **(A)** Effects of caspase inhibition on BPDE-treated cells. HeLa cells were pre-incubated with DMSO or Z-VAD-FMK (20 μM), treated with BPDE (1 μM) and harvested at the indicated time points. The cells were then analyzed for the generation of large DNA fragments and excised oligomers released from genomic DNA. Quantitative analysis of the generation of large DNAs **(B)** is also shown. **(C)** Effects of caspase inhibition on CPT-treated cells. HeLa cells were pre-incubated with DMSO or Z-VAD-FMK (20 μM), treated with CPT (20 μM) and analyzed as in (**A**). A longer exposure of the 20–40 nt region of the blot is provided to show that camptothecin does not induce canonical NER products. Quantitative analysis of the generation of large DNAs is shown in **(D)**.

We next wished to determine whether the large, soluble DNA species that are observed in cells treated with UV mimetic compounds^9^ are also generated in a caspase-dependent manner. As shown in Fig. 6A and quantified in Fig. 6B, the large, soluble DNAs that are produced at late time points after administration with the environmental carcinogen BPDE could be significantly reduced by treatment of the cells with the caspase inhibitor Z-VAD-FMK. However, analyses of the small, excised products of nucleotide excision repair showed that repair was not affected to a significant extent by caspase inhibition.

Camptothecin is a commonly used anti-cancer drug that traps topoisomerase I on the DNA and therefore generates single-strand breaks in DNA^45,46^. Transcription and replication at these sites can convert the single-strand breaks intermediates into potentially lethal double-strand breaks. Using our assay for monitoring soluble DNAs, we observed a robust production of large, soluble DNAs that coincided in time with the cleavage of PARP (Fig. 6C). Moreover, these DNAs were found to be due to apoptotic signaling, as caspase inhibition largely abolished the production of the large, soluble DNAs (Fig. 6C,D). Because camptothecin does not generate a substrate for the nucleotide excision repair machinery, no small DNAs were observed in these experiments. Taken together, our *in vivo* excision repair assay for monitoring the excised oligonucleotide products of excision repair in cultured cells provides not only a high resolution view of repair kinetics but also a sensitive measure of apoptotic signaling, which to our knowledge has not been reported elsewhere.

## Discussion

Here we showed that although apoptotic signaling does not affect the generation or stability of the excised oligonucleotide products of nucleotide excision repair, it does produce a DNA species that can be readily visualized using the same experimental methodology for monitoring the small oligonucleotides products of canonical nucleotide excision repair. Though we have previously reported observing these larger DNA species in cells treated DNA damaging agents^8,9^, their source and mechanism of production remained unknown. The fact that the generation of these DNAs is largely attenuated in the presence of a caspase inhibitor (Fig. 3) or with genetic disruption of caspase 3 (Fig. 5) indicates that they are primarily produced by caspase-mediated apoptotic signaling. Caspase-mediated fragmentation of genomic DNA is considered a biochemical hallmark of apoptosis^47–49^. This cleavage of chromosomal DNA into oligonucleosomal fragments is caused by the activation of endogenous endonucleases such as caspase 3-activated DNase (CAD)^50^, which leads to DNA laddering as multiples of ~180 bp oligonucleosomal size fragments that are visible by agarose gel electrophoresis^49^. The TUNEL (terminal deoxynucleotidyl transferase dUTP nick end labeling) assay is also widely used to detect apoptotic cells; however, it may not discriminate from DNA repair intermediates^51^. Though the use of terminal transferase labeling, urea-PAGE, and chemiluminescent detection, we show here that additional caspase-mediated degradation of DNA down to ~70 nt in length can be readily observed on the same gels that are used to visualize the ~30-nt-long DNA products of nucleotide excision repair. Thus, our ability to now define the origin of the large, soluble DNAs as arising from apoptotic signaling (Figs 3–6) will be useful in future analyses of nucleotide excision repair and other DNA damage responses in both cultured cells and animal tissues. However, we note that there may be some quantitative limitations to the existing assay, and care must be taken to observe repair and apoptotic signals within the linear range of detection. Moreover, terminal transferase may add multiple biotinylated nucleotides to the 3′ ends of the DNAs, which could amplify the observed signals. Because the excised oligonucleotide products of repair in our experiments are of the expected size (20–30 nt), terminal transferase does not appear to be adding multiple nucleotides to these DNAs to any significant extent. In contrast, it is not known whether terminal transferase adds multiple nucleotides to the apoptotic DNAs, and thus it is possible that the large, apoptotic DNA signals that are observed may be artificially amplified by the current methodology.

The ability to detect damage-containing excision products and apoptotic fragments will be useful for biochemical studies addressing their fate in cells and tissues, including in identifying novel proteins that associate with these DNA species to promote their ultimate degradation. UV-irradiated DNA has been shown to less efficiently degraded by cellular nucleases^52^ and thus, if inappropriately localized to the cytosol, these DNAs have the potential to stimulate innate immune signaling pathways that are implicated in autoimmune disorders^21–24^. Thus, in addition to understanding the fate of the canonical excised products of nucleotide excision repair, it will be interesting to determine how cells and tissues process these larger, damage-containing DNAs that are generated during apoptosis.

## Electronic supplementary material

Supplementary Figures
