# Supplementary material for: Simultaneous detection of nucleotide excision repair events and apoptosis-induced DNA fragmentation in genotoxin-treated cells
Source: Sci Rep. 2018 Feb 2;8:2265. doi: 10.1038/s41598-018-20527-6 (PMC5797224; doi:10.1038/s41598-018-20527-6)
Supplement: Supplementary file 1 — Supplementary Figures [file 41598_2018_20527_MOESM1_ESM.docx]

**Simultaneous detection of nucleotide excision repair events and apoptosis-induced DNA fragmentation in genotoxin-treated cells**

Soyun Baek**^§^**^1, 2^, Sueji Han**^§^**^1, 2^, Dukjin Kang^1^, Michael G. Kemp*^3^, and Jun-Hyuk Choi*^1, 2^

^1^Center for Bioanalysis, Korea Research Institute of Standards and Science, Republic of Korea, ^2^Department of Bio-Analytical Science, University of Science & Technology, Republic of Korea, ^3^Department of Pharmacology and Toxicology, Wright State University Boonshoft School of Medicine, Dayton, Ohio

*Co-corresponding authors e-mail: [junchoi@kriss.re.kr](mailto:junchoi@kriss.re.kr) or mike.kemp@wright.edu

^§^These authors contributed equally to this work.

**Supplementary Figure 1. Generation of large DNA fragments in response to UV-mimetic agents.** Quantitiative analysis were performed from previously published data ^1^ that had been obtained from experiments using HeLa cells treated with 1 μM BPDE (A and B) or 0.6 mM cisplatin (C and D).

**Supplementary Figure 2. No effects of caspase inhibition on the protein levels of nucleotide excision repair factors.** UV-irradiated cells in the presence of DMSO or Z-VAD-FMK (20 μM) were harvested at the indicated time points and analyzed by immunoblotting with antibodies against the indicated excision repair proteins.

**Supplementary Figure 3.** Full-length images of representative images presented in cropped format in Figure 1 and 2.

**Supplementary Figure 4.** Full-length images of representative images presented in cropped format in Figure 3.

**Supplementary Figure 5.** Full-length images of representative images presented in cropped format in Supplementary Figure 2.

**Supplementary Figure 6.** Full-length images of representative images presented in cropped format in Figure 5 and 6.

**References**

1 Choi, J. H., Kim, S. Y., Kim, S. K., Kemp, M. G. & Sancar, A. An Integrated Approach for Analysis of the DNA Damage Response in Mammalian Cells: NUCLEOTIDE EXCISION REPAIR, DNA DAMAGE CHECKPOINT, AND APOPTOSIS. *J Biol Chem* **290**, 28812-28821, doi:10.1074/jbc.M115.690354 (2015).
